# Supplementary material for: Moroccan natural products for multitarget-based treatment of Alzheimer’s disease: A computational study
Source: PLoS One. 2025 Jan 16;20(1):e0313411. doi: 10.1371/journal.pone.0313411 (PMC11737685; doi:10.1371/journal.pone.0313411)
Supplement: S1 Table — (DOCX) [file pone.0313411.s001.docx]

Moroccan natural products for multitarget-based treatment of Alzheimer’s disease: A computational study

# Fatima Zahra Guerguer ^(1)^, Amal Bouribab ^(1)^, El Mehdi Karim ^(1)^, Meriem Khedraoui ^(1)^, Fatiha Amegrissi ^(1)^, Yasir S. Raouf ^(2)^, Abdelouahid Samadi ^(2, *)^, and Samir Chtita ^(1, *)^

# Laboratory of Analytical and Molecular Chemistry, Faculty of Sciences Ben M’Sik, Hassan II University of Casablanca, Morocco

# Department of Chemistry, College of Science, United Arab Emirates University, Al Ain P.O. Box 15551, United Arab Emirates

^(*)^ Correspondence: A.S.: [samadi@uaeu.ac.ae](mailto:samadi@uaeu.ac.ae) ; S.C.: [samirchtita@gmail.com](mailto:samirchtita@gmail.com)

Supporting information

**Table S1: 2D structures and detailed Information on the studied compounds**

| Botanical Family | Plant | Compound | No | Structures | Extract | Part of the Plant and Collection Zone |
| --- | --- | --- | --- | --- | --- | --- |
| Amaranthaceae | *Anabasis aretioides* L. | Gallic acid | 1 |  | Cold maceration extract | Aerial parts, Roots and seeds from the region of Errachidia [1] |
|  |  | Catechin | 2 |  |  |  |
|  |  | Rutin | 3 |  |  |  |
|  |  | Chlorogenic acid | 4 |  |  |  |
|  |  | Vanillic acid | 5 |  |  |  |
|  |  | Caffeic acid | 6 |  |  |  |
|  |  | Quercetin | 7 |  |  |  |
|  |  | Syringic acid | 8 |  |  |  |
|  |  | p-hydroxybenzoΪcacid | 9 |  |  |  |
|  |  | Epicatechin | 10 |  |  |  |
|  |  | Coumaric acid | 11 |  |  |  |
|  |  | Salicylic acid | 12 |  |  |  |
|  |  | Catechol | 13 |  |  |  |
|  |  | Pyrogallol | 14 |  |  |  |
|  |  | Ferulic acid | 15 |  |  |  |
|  |  | Naringin | 16 |  |  |  |
|  |  | Hesperidin | 17 |  |  |  |
|  |  | 3-hydroxycinnamic acid | 18 |  |  |  |
|  |  | 4-hydroxycinnamic acid | 11 |  |  |  |
|  |  | 3,4-dihydroxybenzoic acid | 20 |  |  |  |
|  |  | 3-hydroxybenzoic acid | 21 |  |  |  |
|  |  | Luteolin | 22 |  |  |  |
|  |  | Naringenin | 23 |  |  |  |
|  |  | Hesperetin | 24 |  |  |  |
|  | *Haloxylon scoparium* L. | Decanoic acid, methyl ester | 25 |  | Dichloromethane extract | Aerial part and root part in the region of Figuig in Moroccan sahara [2] |
|  |  | Heptandioic acid, dimethyl ester | 26 |  |  |  |
|  |  | 4-Ketopelarrgonic acid methyl ester | 27 |  |  |  |
|  |  | Methyl 8-oxononanoate | 28 |  |  |  |
|  |  | Undecanoic acid, methyl ester | 29 |  |  |  |
|  |  | Nonanoic acid, 9-oxo-, methyl ester | 30 |  |  |  |
|  |  | Octanedioic acid, dimethyl ester | 31 |  |  |  |
|  |  | Dodecanoic acid, methyl ester | 32 |  |  |  |
|  |  | Nonanedioic acid, dimethyl ester | 33 |  |  |  |
|  |  | Tridecanoic acid, methyl ester | 34 |  |  |  |
|  |  | Decanedioic acid, dimethyl ester | 35 |  |  |  |
|  |  | Tetradecanoic acid, methyl ester | 36 |  |  |  |
|  |  | Undecanedioic acid, dimethyl ester | 37 |  |  |  |
|  |  | Pentadecanoic acid, methyl ester | 38 |  |  |  |
|  |  | Dodecanedioic acid, dimethyl ester | 39 |  |  |  |
|  |  | 15-methylhexadecanoic acid, methyl ester | 40 |  |  |  |
|  |  | Hexadecanoic acid, methyl ester | 41 |  |  |  |
|  |  | Heptadecanoic acid, methyl ester | 42 |  |  |  |
|  |  | γ-dodecalactone | 43 |  |  |  |
|  |  | 10,13-octadecadienoic acid, methyl ester | 44 |  |  |  |
|  |  | 9,12-octadecadienoic acid, methyl ester | 45 |  |  |  |
|  |  | 12,15-octadecadienoic acid, methyl ester | 46 |  |  |  |
|  |  | 9,12,15-octadecatrienoic acid, methyl ester | 47 |  |  |  |
|  |  | 13-octadecenoic acid, methyl ester | 48 |  |  |  |
|  |  | 9-octadecenoic acid, methyl ester | 49 |  |  |  |
|  |  | 11-octadecenoic acid, methyl ester | 50 |  |  |  |
|  |  | Octadecanoic acid, methyl ester | 51 |  |  |  |
|  |  | Nonadecanoic acid, methyl ester | 52 |  |  |  |
|  |  | 6,9,15-octadecatrienoic acid, methyl ester | 53 |  |  |  |
|  |  | 6,9,12-octadecatrienoic acid, methyl ester | 54 |  |  |  |
|  |  | 7,10,13-octadecatrienoic acid, methyl ester | 55 |  |  |  |
|  |  | Eicosanoic acid, methyl ester | 56 |  |  |  |
|  |  | Heneicosanoic acid, methyl ester | 57 |  |  |  |
|  |  | Docosanoic acid, methyl ester | 58 |  |  |  |
|  |  | Tricosanoic acid, methyl ester | 59 |  |  |  |
|  |  | Tetracosanoic acid, methyl ester | 60 |  |  |  |
|  |  | Pentacosanoic acid, methyl ester | 61 |  |  |  |
|  |  | Docosanedioic acid, dimethyl ester | 62 |  |  |  |
|  |  | Hexacosanoic acid, methyl ester | 63 |  |  |  |
|  |  | Acetamide, N-methyl-N-(2-phenylethyl)- | 64 |  | Ethyl acetate extract, butanolic extract and aqueous extract. | Aerial part and Root part in the region of Figuig in Moroccan sahara [2] |
|  |  | 4-(2-(N-methylacetamido)ethyl)phenyl acetate | 65 |  |  |  |
|  |  | Acetamide, N-[2-(acetoxy)-2-phenylethyl]- | 66 |  |  |  |
|  |  | 6,7-dimethoxy-4-methylquinoline | 67 |  |  |  |
|  |  | N-(4-Hydroxyphenethyl)acetamide | 68 |  |  |  |
|  |  | 9H-Pyrido[3,4-b]indole, N-acetyl, 1-methyl- | 69 |  |  |  |
|  |  | Acetamide, N-[2-[4-(acetyloxy)−3-methoxyphenyl]ethyl]- | 70 |  |  |  |
|  |  | 3-(2-N-Acetyl-N-methylaminoethyl)indol | 71 |  |  |  |
|  |  | 4-[1-Acetoxy-2-(diacetylamino)ethyl]phenyl acetate | 72 |  |  |  |
|  |  | Tryptamine, 2 acetyl | 73 |  |  |  |
|  |  | 1-Acetylamino-2-(4-acetoxy-3-hydroxyphenyl)ethane | 74 |  |  |  |
|  |  | 1,2,3,4-Tetrahydroisoquinolin, 1-methyl-2-acetyl-6-acetoxy-7‑methoxy- | 75 |  |  |  |
|  |  | Isoquinolin-1-carboxylicacid, 1,2,3,4-tetrahydro-2-acetyl | 76 |  |  |  |
|  |  | N-methyltryptamine, 2 acetyl | 77 |  |  |  |
|  |  | 2-acetyl-1-methyl-1,2,3,4-tetrahydroisoquinoline-6,7-diyl diacetate | 78 |  |  |  |
|  |  | D-glucose, 2,3,4,5,6-pentaacetate | 79 |  |  |  |
|  |  | α-D-Glucopyranose pentaacetate | 80 |  |  |  |
|  |  | alpha-D-Glucopyranoside, 1,3,4,6-tetra-O-acetyl-beta-D-fructofuranosyl, 2,3,4,6-tetraacetate | 81 |  |  |  |
|  |  | 1,1,2-triacetoxyethane | 82 |  |  |  |
|  |  | α-D-Galactose pentaacetate | 83 |  |  |  |
|  |  | Lyxopyranose tetraacetate | 84 |  |  |  |
| Astraceae | *Anacyclus pyrethrum* L. | N-isobutyl-dodeca-2,4,8,10-tetraenamide | 85 |  | Maceration (ethanol exctract) | roots, seeds, leaves, and capitula from the Timahdite region of Morocco [3] |
|  |  | Sarcosine, N-(trifluoroacetyl)-, butyl ester | 86 |  |  |  |
|  |  | N-isobutyl-2,4-octadiene-6- monoynamide | 87 |  |  |  |
|  |  | Levulinic acid | 88 |  |  |  |
|  |  | Propanedioic acid | 89 |  |  |  |
|  |  | N-isobutyl-2,4-heptadiene-6- monoynamide | 90 |  |  |  |
|  |  | Palmitic Acid | 91 |  |  |  |
|  |  | Morphinan-6-One, 4,5.α.-Epoxy-3-Hydroxy-17-Methyl | 92 |  |  |  |
|  |  | Cinnamic acid | 93 |  |  |  |
|  |  | (2E,4E)-N-isobutylundeca-2,4-dien-8,10-diynamide | 94 |  |  |  |
|  |  | 2,4-undecadiene-8,10-diyne-N-tyramide | 151 |  |  |  |
|  |  | N-isobutyl-dodeca-2,4,8,10-tetraenamide (Anacycline) | 95 |  |  |  |
|  |  | N-isobutyl-2,6,8-decatrienamide | 96 |  |  |  |
|  |  | (2E,4E)-N-(2-methylpropyl)deca-2,4-dienamide (Pellitorine) | 103 |  |  |  |
|  |  | Tetradeca-2E-diny-8,10-diynoic acid IBA | 150 |  |  |  |
|  |  | Tetradeca-2E,4E, nE-trienoic-8,10-diynoic acid IBA | 155 |  |  |  |
|  |  | Isovaleric acid | 98 |  |  |  |
|  |  | Dodeca-2E,4E, nE-trienoic acid 4-hydroxyphenylethylamide | 152 |  |  |  |
|  |  | 2,8-N-isobutyl-2,8-dodecadienamide | 210 |  |  |  |
|  |  | Tetradeca-2E,4E,8Etrienoic acid 4-hydroxyphenylethylamide | 153 |  |  |  |
|  |  | Citric acid | 99 |  | Aqueous macerate extract | roots Bin El Ouidan region, Morocco [4] |
|  |  | Gallic acid | 1 |  |  |  |
|  |  | Dihydroxybenzoic acid | 110 |  |  |  |
|  |  | Dihydroxybenzoic acid glucoside | 352 |  |  |  |
|  |  | Caffeoylglucaric acid | 100 |  |  |  |
|  |  | Gallic acid | 1 |  |  |  |
|  |  | 3,4-dihydroxybenzoic acid | 20 |  |  |  |
|  |  | Hydroxybenzoic acid glycerol | 19 |  |  |  |
|  |  | Chlorogenic acid | 4 |  |  |  |
|  |  | Dihydroxybenzoic acid glucuronide | 355 |  |  |  |
|  |  | Pellitorine | 103 |  |  |  |
|  |  | Dihydrocaffeic acid | 104 |  |  |  |
|  |  | Feruloylquinic acid | 105 |  |  |  |
|  |  | Cryptochlorogenic acid | 106 |  |  |  |
|  |  | Caffeic acid | 6 |  |  |  |
|  |  | p-Coumaric acid | 11 |  |  |  |
|  |  | p-Coumaroylquinic acid | 107 |  |  |  |
|  |  | Hydroxycoumarin | 108 |  |  |  |
|  |  | Isochlorogenic acid b | 109 |  |  |  |
|  |  | Quercetin rhamnoside | 110 |  |  |  |
|  |  | Isochlorogenic acid C | 111 |  |  |  |
|  |  | Hexanal | 112 |  | EO | Bensliman in Morocco [5] |
|  |  | α-pinene | 113 |  |  |  |
|  |  | Camphene | 114 |  |  |  |
|  |  | β-Pinene | 115 |  |  |  |
|  |  | Myrcene | 116 |  |  |  |
|  |  | p-cymene | 117 |  |  |  |
|  |  | Limonene | 118 |  |  |  |
|  |  | Linalool | 119 |  |  |  |
|  |  | Nonanol | 120 |  |  |  |
|  |  | Estragole | 121 |  |  |  |
|  |  | α-terpineol | 122 |  |  |  |
|  |  | E-Anethole | 123 |  |  |  |
|  |  | Thymol | 124 |  |  |  |
|  |  | Bornyl acetate | 125 |  |  |  |
|  |  | Carvacrol | 126 |  |  |  |
|  |  | Nerylacetate | 127 |  |  |  |
|  |  | Geranylacetone | 128 |  |  |  |
|  |  | (E)-β-Fernesene | 129 |  |  |  |
|  |  | β-humulene | 130 |  |  |  |
|  |  | Alpha-Muurolene | 131 |  |  |  |
|  |  | Germacrene D | 132 |  |  |  |
|  |  | b-Bisabolene | 133 |  |  |  |
|  |  | Cubebol | 134 |  |  |  |
|  |  | cis-3-Hexenylbenzoate | 135 |  |  |  |
|  |  | Spathulenol | 136 |  |  |  |
|  |  | Caryophylene oxide | 137 |  |  |  |
|  |  | 4(14)-Salvialene-1-one | 138 |  |  |  |
|  |  | Caryophylla-4(14), 8(15)-dien-5-ol | 139 |  |  |  |
|  |  | Vulgarone-B | 140 |  |  |  |
|  |  | α-cadinol | 141 |  |  |  |
|  |  | α-bisabolol | 142 |  |  |  |
|  |  | Phytone | 143 |  |  |  |
|  |  | Caffeic acid | 6 |  | Cold maceration ethanol | leaves, empty capitulas, seeds, and roots from the Timahdite [6] |
|  |  | Hydroxytyrosol | 144 |  |  |  |
|  |  | L-arginine | 279 |  |  |  |
|  |  | Gallic acid | 1 |  |  |  |
|  |  | Pellitorine | 103 |  |  |  |
|  |  | Catechin | 2 |  |  |  |
|  |  | Vanillic acid | 5 |  |  |  |
|  |  | Chlorogenic acid | 4 |  |  |  |
|  |  | Coumarin | 146 |  |  |  |
|  |  | Cinnamic acid | 93 |  |  |  |
|  |  | P-coumaric acid | 11 |  |  |  |
|  |  | Trans ferulic acid | 15 |  |  |  |
|  |  | Oleuropein | 148 |  |  |  |
|  |  | Naringin | 16 |  |  |  |
|  |  | Quercetin | 7 |  |  |  |
|  |  | Geraniol | 149 |  |  |  |
|  |  | Hesperetin | 24 |  |  |  |
|  |  | Deca-2E,4E-dienoic acid N-Me IBA | 154 |  |  |  |
|  |  | Anacyclin | 155 |  |  |  |
|  | *Artemisia mesatlantica* L. | Santolinatriene | 156 |  | EO | The leaves and flowering tops collected in the central Middle Atlas mountain zone [7] |
|  |  | Tricyclene | 157 |  |  |  |
|  |  | α-Thujene | 158 |  |  |  |
|  |  | α-Pinene | 113 |  |  |  |
|  |  | Camphene | 114 |  |  |  |
|  |  | Sabinene | 159 |  |  |  |
|  |  | Β-Pinene | 115 |  |  |  |
|  |  | 1-Decene | 160 |  |  |  |
|  |  | Myrcene | 116 |  |  |  |
|  |  | Yomogi alcohol | 161 |  |  |  |
|  |  | α-Terpinene | 162 |  |  |  |
|  |  | ρ-Cymene | 117 |  |  |  |
|  |  | o-Cymene | 164 |  |  |  |
|  |  | 1,8-Cineole | 165 |  |  |  |
|  |  | Santolina alcohol | 166 |  |  |  |
|  |  | β -(E)-Ocimene | 167 |  |  |  |
|  |  | Bergamal | 168 |  |  |  |
|  |  | Artemisia ketone | 169 |  |  |  |
|  |  | cis-Sabinene hydrate | 170 |  |  |  |
|  |  | cis-Thujone | 171 |  |  |  |
|  |  | trans-Thujone | 226 |  |  |  |
|  |  | neo-Isopulegol | 173 |  |  |  |
|  |  | Chrysanthenone | 174 |  |  |  |
|  |  | allo-Ocimene | 175 |  |  |  |
|  |  | trans-Pinocarveol | 176 |  |  |  |
|  |  | trans-ρ-Menth-2-en-1-ol | 177 |  |  |  |
|  |  | trans-Sabinol | 178 |  |  |  |
|  |  | Camphor | 179 |  |  |  |
|  |  | Pinocarvone | 180 |  |  |  |
|  |  | Borneol | 181 |  |  |  |
|  |  | 4-hexen-1-ol,5-methyl-2-(1-methylethenyl) Lavandulol | 182 |  |  |  |
|  |  | Terpinen-4-ol | 183 |  |  |  |
|  |  | Thuj-3-en-10-al | 184 |  |  |  |
|  |  | Prenylangelate | 367 |  |  |  |
|  |  | Myrtenal | 185 |  |  |  |
|  |  | Myrtenol | 186 |  |  |  |
|  |  | γ-Terpineol | 187 |  |  |  |
|  |  | trans-Piperitol | 188 |  |  |  |
|  |  | endo-Fenchyl acetate | 189 |  |  |  |
|  |  | Piperitone | 190 |  |  |  |
|  |  | cis-Chrysanthenylacetate | 191 |  |  |  |
|  |  | Isobornylacetate | 192 |  |  |  |
|  |  | exo-Arbozol | 330 |  |  |  |
|  |  | Sesquicineole<7-epi-1,2-dehydro-> | 337 |  |  |  |
|  |  | Germacrene D | 132 |  |  |  |
|  |  | Spathulenol | 136 |  |  |  |
|  |  | Globulol | 193 |  |  |  |
|  |  | Viridiflorol | 194 |  |  |  |
|  |  | Eremoligenol | 195 |  |  |  |
|  |  | α-Cadinol | 141 |  |  |  |
|  |  | Botrydiol | 229 |  |  |  |
|  | *Artemisia vulgaris* L. | Camphene | 114 |  | EO | leaves from different locations in Morocco [8] |
|  |  | 3,3,6-trimethyl-1,4-heptadien-6-ol | 196 |  |  |  |
|  |  | o-cymol | 164 |  |  |  |
|  |  | Cineole | 165 |  |  |  |
|  |  | 4-carene | 215 |  |  |  |
|  |  | butyric acid, 3-hexenyl ester | 199 |  |  |  |
|  |  | 2,7-dimetyl-2,6-octadien-4-ol | 200 |  |  |  |
|  |  | β,-thujone | 172 |  |  |  |
|  |  | α,-thujone | 201 |  |  |  |
|  |  | Fenchene | 202 |  |  |  |
|  |  | trans-Pinocarveol | 176 |  |  |  |
|  |  | Camphor | 179 |  |  |  |
|  |  | 3-pinanone | 203 |  |  |  |
|  |  | Santolina triene | 156 |  |  |  |
|  |  | Borneol | 181 |  |  |  |
|  |  | α-lonol | 310 |  |  |  |
|  |  | 4-terpineol | 183 |  |  |  |
|  |  | Myrtenol | 186 |  |  |  |
|  |  | isopinocampheol | 239 |  |  |  |
|  |  | 6-(1-butenyl)-1,4-cycloheptadiene | 208 |  |  |  |
|  |  | α,-limonene diepoxide | 246 |  |  |  |
|  |  | Piperitone | 190 |  |  |  |
|  |  | Chrysanthenyl acetate | 242 |  |  |  |
|  |  | 3-carene, 2-(acetylmethyl) | 213 |  |  |  |
|  |  | Bornyl acetate | 125 |  |  |  |
|  |  | 6-hexadecen-4-yne | 212 |  |  |  |
|  |  | Copaene | 335 |  |  |  |
|  |  | Caryophyllene | 214 |  |  |  |
|  |  | Gamma-muurolene | 217 |  |  |  |
|  |  | Germacrene D | 132 |  |  |  |
|  |  | Gamma-Elemene | 216 |  |  |  |
|  |  | α-muurolene | 131 |  |  |  |
|  |  | Delta-cadinene | 346 |  |  |  |
|  |  | Spathulenol | 136 |  |  |  |
|  |  | Carotol | 219 |  |  |  |
|  | *Artemisia ifranensis* L. | α-Pinene | 113 |  | Essential oils | Leaves from Timahdite |
|  |  | β-Pinene | 115 |  |  |  |
|  |  | Cymene | 117 |  |  |  |
|  |  | Cineole | 165 |  |  |  |
|  |  | Terpinene | 183 |  |  |  |
|  |  | Sabinene hydtrate | 97 |  |  |  |
|  |  | Linalool oxide | 299 |  |  |  |
|  |  | Trans-Sabinenehydrate | 365 |  |  |  |
|  |  | Thujone trans | 227 |  |  |  |
|  |  | Thujone cis | 172 |  |  |  |
|  |  | Fenchol | 228 |  |  |  |
|  |  | Thujanol <iso-3> | 204 |  |  |  |
|  |  | Sabinol | 178 |  |  |  |
|  |  | Terpinen-4-ol | 183 |  |  |  |
|  |  | Terpineol | 122 |  |  |  |
|  |  | Myrtenol | 186 |  |  |  |
|  |  | Dihydrocarveol< iso> | 348 |  |  |  |
|  |  | Fragranol | 356 |  |  |  |
|  |  | Dihydrocarveol | 235 |  |  |  |
|  |  | Sabinyl acetate | 234 |  |  |  |
|  |  | Dihydrocarveol acetate | 237 |  |  |  |
|  |  | Caryophylene<E> | 214 |  |  |  |
|  |  | Germacrene-D | 240 |  |  |  |
|  |  | Ionone<methyl-y> | 366 |  |  |  |
|  |  | Cubebol | 134 |  |  |  |
|  |  | Italicene epoxide | 369 |  |  |  |
|  |  | Patchouli alcool | 243 |  |  |  |
|  |  | Palustrol | 236 |  |  |  |
|  |  | Dendrolasin | 245 |  |  |  |
|  |  | Spathulenol | 136 |  |  |  |
|  |  | Caryophyllene oxide | 137 |  |  |  |
|  |  | Salvial-4(14)-en-1-one | 247 |  |  |  |
|  |  | Guaiol | 248 |  |  |  |
|  |  | Cedrol | 249 |  |  |  |
|  |  | Eremoligenol | 195 |  |  |  |
|  |  | Caryophylla-4(12),8(13)-dien-5β-ol | 250 |  |  |  |
|  |  | Hinesol | 251 |  |  |  |
|  |  | Khusimol | 252 |  |  |  |
|  |  | α-Cadinol | 141 |  |  |  |
|  |  | Patchouli alcohol | 243 |  |  |  |
|  |  | Bisabolol oxide B | 253 |  |  |  |
|  |  | Isobornyl isobutanoate | 254 |  |  |  |
|  |  | Valeranone | 255 |  |  |  |
|  |  | Guaia-3,10(14)-dien-11-ol | 256 |  |  |  |
|  |  | Bisabolone oxide A | 257 |  |  |  |
|  |  | Curcumen-12-ol | 258 |  |  |  |
|  |  | Cedr-8(15)-en-9- α -ol acetate | 259 |  |  |  |
|  |  | Bisabolol oxide-A | 260 |  |  |  |
|  |  | Lanceol | 261 |  |  |  |
|  |  | Cedryl acetate | 262 |  |  |  |
|  |  | Cedren-13-ol acetate | 263 |  |  |  |
|  |  | Chenopodiol | 264 |  |  |  |
| Fabaceae | *Vicia faba* L. | 4-hydroxyphenyl acetic acid | 265 |  | Olive mill waste water | olive mill in Marrakech [9] |
|  |  | Gallic acid | 1 |  |  |  |
|  |  | veratric acid | 266 |  |  |  |
|  |  | paracoumaric acid | 11 |  |  |  |
|  |  | Caffeic acid | 6 |  |  |  |
|  |  | Oleuropein | 148 |  |  |  |
| Lamiaceae | *Ajuga iva* L. | Abutasterone | 267 |  | Ethanolic extract | Aerial parts (Morocco) Taroudant [10] |
|  |  | Cyasterone | 268 |  |  |  |
|  |  | 24-hydroxycyasterone | 269 |  |  |  |
|  |  | 22-Dehydrocyasterone 2-glucoside | 270 |  |  |  |
|  |  | 20-Hydroxyecdysone | 271 |  |  |  |
|  |  | Makisterone A | 272 |  |  |  |
|  |  | Ponasterone A | 273 |  |  |  |
|  |  | Sidisterone | 274 |  |  |  |
|  |  | Galacturonic acid | 275 |  | Decoction Extracts | leaves from the Masmouda region of Morocco 17 |
|  |  | Myricetin | 276 |  |  |  |
|  |  | Gallic acid | 1 |  |  |  |
|  |  | Ascorbic acid | 277 |  |  |  |
|  |  | Mucic acid | 278 |  |  |  |
|  |  | Arginine | 279 |  |  |  |
|  |  | Vanillin | 280 |  |  |  |
|  |  | Quinic acid | 281 |  |  |  |
|  |  | Cinnamic acid | 93 |  |  |  |
|  |  | Rhamnetin | 282 |  |  |  |
|  |  | Catechin-7-O-glucoside | 283 |  |  |  |
|  |  | Catechin | 2 |  |  |  |
|  |  | Harpagid | 284 |  |  |  |
|  |  | Ajugasterone D | 285 |  |  |  |
|  |  | Coumarin | 146 |  |  |  |
|  |  | Ferulic acid 4-O-glucoside | 286 |  |  |  |
|  |  | Coumaric acid | 11 |  |  |  |
|  |  | Epigallocatechin gallate | 288 |  |  |  |
|  |  | 8-O-acetyl-harpagid | 289 |  |  |  |
|  |  | Ferulic acid | 15 |  |  |  |
|  |  | Cholesterol | 290 |  |  |  |
|  |  | Cyasterone | 268 |  |  |  |
|  |  | Resveratrol 3-Glucoside | 291 |  |  |  |
|  |  | Apigenin-7-(2-O-apiosylglucoside) | 292 |  |  |  |
|  |  | Apigenin 7-O-(6″-malonyl-apiosyl-glucoside) | 293 |  |  |  |
|  |  | Apigenin | 294 |  |  |  |
|  |  | Quercetin-3-O-pentosyl-pentoside | 295 |  |  |  |
|  |  | Quercetin | 7 |  |  |  |
|  |  | Kaempferide | 296 |  |  |  |
|  |  | Luteolin | 22 |  |  |  |
|  |  | Trans-p-coumaric acid | 11 |  |  |  |
|  |  | Vanillic acid glucoside | 298 |  |  |  |
|  | *Lavandula angustifolia* L. | cis-linalool oxide | 299 |  | EO | collected in the rural municipality of Talsint, situated in Figuig-Morocco [11] |
|  |  | Camphene | 114 |  |  |  |
|  |  | β-Pinene | 115 |  |  |  |
|  |  | trans-linalool oxide | 300 |  |  |  |
|  |  | p-cymene | 117 |  |  |  |
|  |  | 1,8-Cineole | 165 |  |  |  |
|  |  | Camphor | 179 |  |  |  |
|  |  | Terpinen-4-ol | 183 |  |  |  |
|  |  | Caryophyllene | 214 |  |  |  |
|  |  | Linalool | 119 |  |  |  |
|  |  | Borneol | 181 |  |  |  |
|  |  | linalyl acetate | 301 |  |  |  |
|  |  | (+)-Epi-bicyclosesquiphellandrene | 302 |  |  |  |
|  | *Lavandula maroccana* L*.* | Furfural | 303 |  | EO | Aerial parts from Ijoukak region [12] |
|  |  | 5-hydroxymethyl furfural | 304 |  |  |  |
|  |  | 1-Octen-3-ol | 305 |  |  |  |
|  |  | 1,8-Cineole | 165 |  |  |  |
|  |  | α -Terpinene | 162 |  |  |  |
|  |  | Linalool | 119 |  |  |  |
|  |  | Terpinen-4-ol | 183 |  |  |  |
|  |  | p-Cymen-8-ol | 308 |  |  |  |
|  |  | α-Terpineol | 122 |  |  |  |
|  |  | 2.3-Dihydrobenzofuran | 309 |  |  |  |
|  |  | Isopiperitone | 190 |  |  |  |
|  |  | Thymol | 124 |  |  |  |
|  |  | Carvacrol | 126 |  |  |  |
|  |  | 2-Methoxy-4-vinylphenol | 311 |  |  |  |
|  |  | α -Terpinyl acetate | 312 |  |  |  |
|  |  | Methyleugenol | 313 |  |  |  |
|  |  | β-Caryophyllene | 314 |  |  |  |
|  |  | α-Farnesene | 297 |  |  |  |
|  |  | Spathulenol | 136 |  |  |  |
|  |  | Caryophyllene oxide | 137 |  |  |  |
|  | *L,pedunculata* L. *L,angustifolia* L. *L,maroccana* L. | Eucalyptol | 165 |  | EO | Aerial parts Khenifra, Ifrane and Volubilis respectively [13] |
|  |  | Fenchone | 316 |  |  |  |
|  |  | 2,4-Di-tertbutylphenol | 317 |  |  |  |
|  | *Mentha suaveolens* L. | Quinic acid | 281 |  | Methanol extract | The aerial parts from middle Atlas of Morocco (Ifrane) [14] |
|  |  | THDBCHMCA: 1,2,6,7-tetrahydroxy-5H-dibenzo- [a,d]cycloheptene-5-methyl-11-carboxylic acid | 318 |  |  |  |
|  |  | Luteolin-dihexoside | 319 |  |  |  |
|  |  | Luteolin-hexoside | 320 |  |  |  |
|  |  | Luteolin-glucuronide | 321 |  |  |  |
|  |  | Apigenin-dihexoside | 322 |  |  |  |
|  |  | Apigenin-glucuronide | 323 |  |  |  |
|  |  | Salvianolic acid B | 324 |  |  |  |
|  |  | Rosmarinic acid | 325 |  |  |  |
|  |  | Dihydroxy-tetramethoxyflavone | 327 |  |  |  |
|  |  | Salvianolic acid A | 326 |  |  |  |
|  |  | Jaceosidin | 329 |  |  |  |
|  |  | Salvianolic acid B/E | 378 |  |  |  |
|  |  | 4-*O*-caffeoylquinic acid | 106 |  | Hydromethanolic extract | leaves and flowers from Taounate [15] |
|  |  | 5-*O*-caffeoylquinic acid | 331 |  |  |  |
|  | *Syzygium aromaticum* L. | Eugenol | 332 |  | EO | stems, leaves and flowers in Morocco [16] |
|  |  | Caryophyllene | 238 |  |  |  |
|  |  | 1,1,4,8-tetramethyl-cis,4,7,10-cycloundecatriene | 333 |  |  |  |
|  |  | Caryophyllene oxide | 137 |  |  |  |
|  |  | Humulene epoxide II | 334 |  |  |  |
|  |  | Aromandendrene | 335 |  |  |  |
|  |  | 2-Pentadecen-4-yne, (Z)- | 336 |  |  |  |
|  |  | δ-Cadinene | 346 |  |  |  |
|  |  | trans-Calamenene | 338 |  |  |  |
|  |  | 1-(3,4-methylenedioxyphenyl)propane-1-ol | 339 |  |  |  |
|  |  | Estragole | 121 |  |  |  |
| Pinaceae | *Pinus halepensis* L. | α-Pinene | 113 |  | EO | needles of Aleppo pine tree from the park Tazekka, in Taza-Region Fez-Meknes (Morocco) [17] |
|  |  | 1R-α-Pinene | 209 |  |  |  |
|  |  | Sabinene | 159 |  |  |  |
|  |  | β-Pinene | 115 |  |  |  |
|  |  | Trans-β-Ocimene | 167 |  |  |  |
|  |  | 4-Isopropylidene-1- cyclohexene | 223 |  |  |  |
|  |  | Copaene | 341 |  |  |  |
|  |  | Caryophyllene | 214 |  |  |  |
|  |  | α-Bisabolene | 342 |  |  |  |
|  |  | α-Humulene | 343 |  |  |  |
|  |  | Isovalerate de β-phenylethy | 344 |  |  |  |
|  |  | α-muurolene | 131 |  |  |  |
|  |  | α-Cadinene | 345 |  |  |  |
|  |  | Caryophyllene oxide | 137 |  |  |  |
|  |  | Guaiol | 248 |  |  |  |
|  |  | Caryophyllene-(I1) | 214 |  |  |  |
|  |  | Cembrene | 241 |  |  |  |
|  |  | Cembrene A | 347 |  |  |  |
|  |  | Thunbergol | 147 |  |  |  |
| [Apiacée](https://www.google.com/search?sca_esv=5480f011bd3e2bf8&sxsrf=ACQVn0-LW2ZEc4gOEstzxKK0DIH_c1UPhw:1713709932155&q=Apiac%C3%A9es&stick=H4sIAAAAAAAAAONgVmLXz9U3yDEzf8RoxC3w8sc9YSnNSWtOXmNU5uIKzsgvd80rySypFBLlYoOyuKU4uWB6eBaxcjoWZCYmH16ZWgwAYr2zf0wAAAA&sa=X&ved=2ahUKEwiy54P-wtOFAxXlUqQEHSPRAHoQzIcDKAB6BAgWEAE) | *Coriandrum sativum* L. | α –Thujene | 158 |  | OE | seeds[18] |
|  |  | Sabinene | 340 |  |  |  |
|  |  | α-Phellandrene | 349 |  |  |  |
|  |  | α-Terpinene | 162 |  |  |  |
|  |  | o-Cymene | 164 |  |  |  |
|  |  | D-Limonene | 371 |  |  |  |
|  |  | Eucalyptol | 165 |  |  |  |
|  |  | (Z)-β-ocimene | 350 |  |  |  |
|  |  | Gamma-Terpinene | 351 |  |  |  |
|  |  | Cis-sabinene | 307 |  |  |  |
|  |  | p mentha-1,4 (8) -dene | 353 |  |  |  |
|  |  | Spiro[4.5]dec-6-ene | 354 |  |  |  |
|  |  | Camphor | 179 |  |  |  |
|  |  | Pinocarvone | 180 |  |  |  |
|  |  | Borneol | 181 |  |  |  |
|  |  | Terpinen-4-ol | 183 |  |  |  |
|  |  | p Cymen-8-ol | 308 |  |  |  |
|  |  | p-menth-1-en-8-ol | 122 |  |  |  |
|  |  | (-)-myrtenol | 101 |  |  |  |
|  |  | Decanal | 357 |  |  |  |
|  |  | (-)-Verbenone | 358 |  |  |  |
|  |  | Citronellol | 315 |  |  |  |
|  |  | Nerol | 359 |  |  |  |
|  |  | Geranial(citral) | 360 |  |  |  |
|  |  | Cuminaldehyde | 361 |  |  |  |
|  |  | Geraniol | 149 |  |  |  |
|  |  | Methyl citronellate | 362 |  |  |  |
|  |  | 2-Decenal, (E)- | 363 |  |  |  |
|  |  | 2-Decen-1-ol | 364 |  |  |  |
|  |  | 1-Decanol | 197 |  |  |  |
|  |  | Anethole | 123 |  |  |  |
|  |  | p-Thymol | 220 |  |  |  |
|  |  | Undecanal | 145 |  |  |  |
|  |  | myrtenyl acetate | 368 |  |  |  |
|  |  | Citronellyl Acetate | 328 |  |  |  |
|  |  | Neryl Acetate | 370 |  |  |  |
|  |  | Dodecanal | 372 |  |  |  |
|  |  | β-Caryophyllene | 314 | C/C/1=C\CCC(=C)[C@H]2CC([C@@H]2CC1)(C)C |  |  |
|  |  | Trans-2-dodecenal | 373 |  |  |  |
|  |  | 2-decylfuran | 374 |  |  |  |
|  |  | Trans-2-Undecen-1-ol | 375 |  |  |  |
|  |  | Myristicin | 376 |  |  |  |
|  |  | Caryophyllene oxide | 137 |  |  |  |
|  |  | gamma-muurolene | 217 |  |  |  |
|  |  | Phytone | 143 |  |  |  |
|  | *Foeniculum vulgare* L. | α-pinene | 113 |  | EO | seeds in the region of Meknes [19] |
|  |  | Camphene | 114 |  |  |  |
|  |  | β-Phellandrene | 377 |  |  |  |
|  |  | β-pinene | 115 |  |  |  |
|  |  | β-myrcene | 116 |  |  |  |
|  |  | α-Phellandrene | 349 |  |  |  |
|  |  | р-Cymene | 117 |  |  |  |
|  |  | D-Limonene | 371 |  |  |  |
|  |  | Eucalyptol | 165 |  |  |  |
|  |  | Bicyclo[3.1.1]hept-2-ene, 3,6,6-trimethyl- | 379 |  |  |  |
|  |  | γ-Terpinen | 351 |  |  |  |
|  |  | L-Fenchone | 232 |  |  |  |
|  |  | Camphor | 179 |  |  |  |
|  |  | Terpinene-4-ol | 183 |  |  |  |
|  |  | Estragole | 121 |  |  |  |
|  |  | Bicyclo[2.2.1]heptan-2-ol, 1,3,3-trimethyl-, acetate, (1S-exo)- | 189 |  |  |  |
|  |  | p-Anisaldehyde | 383 |  |  |  |
|  |  | 1-Butanone, 2-chloro-3-methyl-1-[4-(1-methylethyl)phenyl]- | 102 |  |  |  |
|  |  | 4-[(S)-sec-Butyl]anisole | 385 |  |  |  |
|  |  | 4-Methoxyphenylacetone | 386 |  |  |  |
|  |  | Formic acid, 2-isopropylphenyl ester | 382 |  |  |  |
|  |  | m-Anisic acid, 4-chlorophenyl ester | 381 |  |  |  |
|  |  | Myristicin | 376 |  |  |  |
|  |  | 1,3-Benzenediamine, N,N,N',N'-tetramethyl | 380 |  |  |  |
|  |  | p-Cymene | 117 |  | EO | The seeds collected from Gourrama, Southeastern Morocco [20] |
|  |  | Limonene | 118 |  |  |  |
|  |  | 3-Carène | 211 |  |  |  |
|  |  | Eucalyptol | 165 |  |  |  |
|  |  | ϒ-Terpinène | 351 |  |  |  |
|  |  | Fenchone | 316 |  |  |  |
|  |  | Carveol | 231 |  |  |  |
|  |  | Camphor | 179 |  |  |  |
|  |  | 4-Terpineol | 183 |  |  |  |
|  |  | Estragole | 121 |  |  |  |
|  |  | p-cumic aldehyde | 361 |  |  |  |
|  |  | Cis-Anethole | 244 |  |  |  |
|  |  | para-Anisaldehyde | 383 |  |  |  |
|  |  | Anethole | 123 |  |  |  |
|  |  | α-Terpinen-7-ol | 206 |  |  |  |
|  | *Pimpinella anisum L* | Camphene | 114 |  | EO | Anise seeds was collected from the middle of Morocco (Meknes) [21] |
|  |  | Limonene | 118 |  |  |  |
|  |  | Fenchone | 316 |  |  |  |
|  |  | 4-allylanisole | 121 |  |  |  |
|  |  | Anethole | 123 |  |  |  |
|  |  | Acide linoléique | 225 |  |  |  |
|  |  | *α*–Pinene | 113 |  | EO | seeds of green anise from taza [22] |
|  |  | Camphene | 114 |  |  |  |
|  |  | Fenchone | 316 |  |  |  |
|  |  | *α*–Campholene | 198 |  |  |  |
|  |  | Fenchyl acetate | 384 |  |  |  |
|  |  | Cis-limonene oxide | 205 |  |  |  |
|  |  | Cis-anethole | 246 |  |  |  |
|  |  | isobornyl acetate | 207 |  |  |  |
|  |  | Trans-anethole | 123 |  |  |  |
|  |  | *α*-Caryophyllene | 343 |  |  |  |
|  |  | Azulene | 218 |  |  |  |
|  |  | Ledene | 221 |  |  |  |
|  | *Apium graveolens* L*. Coriandrum sativum* L. *Petroselinum crispum* L*.* | Caffeic acid | 6 |  | Methanol extract | The  celery, coriander, and parsley were collected during the flowering period from the Tinghir region located in the southeast of Morocco [23] |
|  |  | Chlorogenic acid | 4 |  |  |  |
|  |  | p-Coumaric acid | 11 |  |  |  |
|  |  | Ferulic acid | 15 |  |  |  |
|  |  | Gallic acid | 1 |  |  |  |
|  |  | Syringic acid | 8 |  |  |  |
|  |  | Vanillic acid | 5 |  |  |  |
|  |  | Luteolin | 22 |  |  |  |
|  |  | Quercetin | 7 |  |  |  |
|  |  | Rutin | 3 |  |  |  |
|  | *Apium Graveolens* L*.* | α-Thujene | 158 |  | Essential oil | Celery Seed in the area of Marrakech [24] |
|  |  | Hydroxybutric acid lactone | 222 |  |  |  |
|  |  | β-Pinene | 115 |  |  |  |
|  |  | Bèta-Myrcene | 116 |  |  |  |
|  |  | D-Limonene | 371 |  |  |  |
|  |  | 6-butyl-1,4-cycloheptadiène | 224 |  |  |  |
|  |  | Amyl benzene | 233 |  |  |  |
|  |  | β-Selinene | 248 |  |  |  |
|  |  | Caryophylene oxide | 137 |  |  |  |
|  |  | beta-Selinenol | 287 |  |  |  |
|  |  | 2-chloro-1-(-2,4-dimethylphenyl)-2-methyl-1-propanone | 306 |  |  |  |
|  |  | α-Hydroxypropylbenzene | 85 |  |  |  |
|  | *Carum carvi* L. | Ferulic acid | 15 |  | maceratio (methenol exctract) | seedsthe region of Taounate (Fez, Morocco) [25] |
|  |  | Gallic acid | 1 |  |  |  |
|  |  | Myricetin | 276 |  |  |  |
|  |  | Catechin | 2 |  |  |  |
|  |  | Caffeic acid | 6 |  |  |  |
|  |  | Quercetin | 7 |  |  |  |
|  | *Petroselinum crispum* L. | Vanillic acid | 5 |  | Soxhle | The leaves of P. crispum were collected in the North of Morocco in April 2016 [26] |
|  |  | Luteolin | 22 |  |  |  |
|  |  | kaempferol | 163 |  |  |  |
|  |  | Quercetin | 7 |  |  |  |
|  |  | apigenin | 294 |  |  |  |

[1] A. Berrani *et al.*, ‘Anabasis aretioides Coss. & Moq. phenolic compounds exhibit in vitro hypoglycemic, antioxidant and antipathogenic properties’, *Journal of Basic and Clinical Physiology and Pharmacology*, vol. 30, no. 2, pp. 251–257, Mar. 2019, doi: 10.1515/jbcpp-2018-0154.

[2] S. Haida, A. Kribii, and A. Kribii, ‘Chemical composition, phenolic content and antioxidant capacity of *Haloxylon scoparium* extracts’, *South African Journal of Botany*, vol. 131, pp. 151–160, Jul. 2020, doi: 10.1016/j.sajb.2020.01.037.

[3] F. Z. Jawhari *et al.*, ‘Anacyclus pyrethrum (L): Chemical Composition, Analgesic, Anti-Inflammatory, and Wound Healing Properties’, *Molecules*, vol. 25, no. 22, Art. no. 22, Jan. 2020, doi: 10.3390/molecules25225469.

[4] A. Baslam *et al.*, ‘Phytochemistry, Antioxidant Potential, and Antibacterial Activities of Anacyclus pyrethrum: Promising Bioactive Compounds’, *Horticulturae*, vol. 9, no. 11, Art. no. 11, Nov. 2023, doi: 10.3390/horticulturae9111196.

[5] K. E. Mokhtari, M. E. Kouali, M. Talbi, L. Hajji, and A. E. Brouzi, ‘Chemical composition and insecticidal activity of Anacyclus pyrethrum essential oil from the Bensliman area against Culex pipiens’, *Mediterranean Journal of Chemistry*, vol. 10, no. 1, Art. no. 1, Jan. 2020, doi: 10.13171/mjc101020211198kem.

[6] F. Z. Jawhari *et al.*, ‘Phytochemical, Morphological and Genetic Characterisation of Anacyclus pyrethrum var. depressus (Ball.) Maire and Anacyclus pyrethrum var. pyrethrum (L.) Link’, *Molecules*, vol. 28, no. 14, Art. no. 14, Jan. 2023, doi: 10.3390/molecules28145378.

[7] S. Amine *et al.*, ‘Influence of Abiotic Factors on the Phytochemical Profile of Two Species of Artemisia: A. herba alba Asso and A. mesatlantica Maire’, *International Journal of Plant Biology*, vol. 13, no. 2, Art. no. 2, Jun. 2022, doi: 10.3390/ijpb13020007.

[8] I. Sbai *et al.*, ‘Chemical composition and antioxidant activity of the essential oil of Artemisia vulgaris from Morocco’, Nov. 2019.

[9] H. El Hajjouji, E. Pinelli, M. Guiresse, G. Merlina, J.-C. Revel, and M. Hafidi, ‘Assessment of the genotoxicity of olive mill waste water (OMWW) with the *Vicia faba* micronucleus test’, *Mutation Research/Genetic Toxicology and Environmental Mutagenesis*, vol. 634, no. 1, pp. 25–31, Dec. 2007, doi: 10.1016/j.mrgentox.2007.05.015.

[10] A. Bouyahya *et al.*, ‘Ethnomedicinal use, phytochemistry, pharmacology, and toxicology of *Ajuga iva* (L.,) schreb’, *Journal of Ethnopharmacology*, vol. 258, p. 112875, Aug. 2020, doi: 10.1016/j.jep.2020.112875.

[11] S. El Kharraf, M. L. Faleiro, F. Abdellah, S. El-Guendouz, E. M. El Hadrami, and M. G. Miguel, ‘Simultaneous Hydrodistillation-Steam Distillation of Rosmarinus officinalis, Lavandula angustifolia and Citrus aurantium from Morocco, Major Terpenes: Impact on Biological Activities’, *Molecules*, vol. 26, no. 18, Art. no. 18, Jan. 2021, doi: 10.3390/molecules26185452.

[12] B. Soulaimani *et al.*, ‘Chemical composition, antimicrobial activity and synergistic potential of essential oil from endemic *Lavandula maroccana* (Mill.)’, *South African Journal of Botany*, vol. 125, pp. 202–206, Sep. 2019, doi: 10.1016/j.sajb.2019.07.030.

[13] A. Nafis *et al.*, ‘Chemical composition and synergistic effect of three Moroccan lavender EOs with ciprofloxacin against foodborne bacteria: a promising approach to modulate antimicrobial resistance’, *Letters Applied Microbiology*, vol. 72, no. 6, pp. 698–705, Jun. 2021, doi: 10.1111/lam.13460.

[14] A. Bouymajane *et al.*, ‘Phenolic Compounds, Antioxidant and Antibacterial Activities of Extracts from Aerial Parts of Thymus zygis subsp. gracilis, Mentha suaveolens and Sideritis incana from Morocco’, *Chemistry & Biodiversity*, vol. 19, no. 3, p. e202101018, 2022, doi: 10.1002/cbdv.202101018.

[15] J. El-Akhal, A. P. Oliveira, R. Bencheikh, P. Valentão, P. B. Andrade, and M. Morato, ‘Vasorelaxant Mechanism of Herbal Extracts from Mentha suaveolens, Conyza canadensis, Teucrium polium and Salvia verbenaca in the Aorta of Wistar Rats’, *Molecules*, vol. 27, no. 24, Art. no. 24, Jan. 2022, doi: 10.3390/molecules27248752.

[16] A. Ainane *et al.*, ‘Chemical composition and insecticidal activity of five essential oils: Cedrus atlantica, Citrus limonum, Rosmarinus officinalis, Syzygium aromaticum and Eucalyptus globules’, *Materials Today: Proceedings*, vol. 13, pp. 474–485, Jan. 2019, doi: 10.1016/j.matpr.2019.04.004.

[17] ‘The Needles of Aleppo Pine From the Province of … — Library of Science’. Accessed: Mar. 01, 2024. [Online]. Available: https://bibliotekanauki.pl/articles/2202321

[18] K. A. Amrani, M. Barbouchi, M. Elidrissi, A. Amechrouq, and M. Chokrad, ‘Chemical composition and physicochemical properties of the essential oil of coriander (Coriandrum sativum L.) grown in Morocco’, *RHAZES: Green and Applied Chemistry*, vol. 4, no. 4, Art. no. 4, May 2019, doi: 10.48419/IMIST.PRSM/rhazes-v4.16202.

[19] M. Barrahi *et al.*, ‘Chemical composition and evaluation of antibacterial activity of fennel (Foeniculum vulgare Mill) seed essential oil against some pathogenic bacterial strains’, *Caspian Journal of Environmental Sciences*, vol. 18, no. 4, pp. 295–307, Oct. 2020, doi: 10.22124/cjes.2020.4276.

[20] M. Abdellaoui, E. dine T. Bouhlali, M. Derouich, and L. El-Rhaffari, ‘Essential oil and chemical composition of wild and cultivated fennel (*Foeniculum vulgare* Mill.): A comparative study’, *South African Journal of Botany*, vol. 135, pp. 93–100, Dec. 2020, doi: 10.1016/j.sajb.2020.09.004.

[21] A. A. Maofari *et al.*, ‘CHEMICAL COMPOSITION AND ANTIBACTERIAL PROPERTIES OF ESSENTIAL OILS OF Pimpinella Anisum L. GROWING IN MOROCCO AND YEMEN’, 2013.

[22] Y. Moustakime, Z. Hazzoumi, and K. Amrani Joutei, ‘Aromatization of virgin olive oil by seeds of *Pimpinella anisum* using three different methods: Physico-chemical change and thermal stability of flavored oils’, *Grain & Oil Science and Technology*, vol. 4, no. 3, pp. 108–124, Sep. 2021, doi: 10.1016/j.gaost.2021.07.001.

[23] M. Derouich, E. D. T. Bouhlali, M. Bammou, A. Hmidani, K. Sellam, and C. Alem, ‘Bioactive Compounds and Antioxidant, Antiperoxidative, and Antihemolytic Properties Investigation of Three *Apiaceae* Species Grown in the Southeast of Morocco’, *Scientifica*, vol. 2020, pp. 1–10, Sep. 2020, doi: 10.1155/2020/3971041.

[24] A. E. Mostaphi, H. E. Hartiti, M. Barrahi, A. Zarrouk, M. Berrabeh, and M. Ouhssine, ‘Etude Physico-chimiques et Analyses Chromatographiques de l’huile Essentiel des Grains de céleri (Apium Graveolens.L) [Physico-chemical and Chromatographic Analysis Study of the Essential oil of Celery Seed (Apium Graveolens.L)]’.

[25] I. Es-safi *et al.*, ‘An Insight into the Anxiolytic and Antidepressant-Like Proprieties of Carum carvi L. and Their Association with Its Antioxidant Activity’, *Life*, vol. 11, no. 3, Art. no. 3, Mar. 2021, doi: 10.3390/life11030207.

[26] J. Kouar *et al.*, ‘Comparison between electrocoagulation and solvent extraction method in the process of the dechlorophyllation of alcoholic extracts from Moroccan medicinal plants Petroselinum crispum, Thymus satureioides and microalgae Spirulina platensis’, *SN Appl. Sci.*, vol. 1, no. 1, p. 132, Jan. 2019, doi: 10.1007/s42452-018-0137-1.
