# Supplementary material for: Moroccan natural products for multitarget-based treatment of Alzheimer’s disease: A computational study
Source: PLoS One. 2025 Jan 16;20(1):e0313411. doi: 10.1371/journal.pone.0313411 (PMC11737685; doi:10.1371/journal.pone.0313411)
Supplement: Attachment — Submitted filename: Review_PONE-D-24-36446.pdf [file pone.0313411.s002.pdf]

Moroccan natural products for multitarget-based treatment of Alzheimer's disease: A computational study involving molecular docking, ADMET analysis, density functional theory, and molecular dynamics simulations

The title is too long! The authors should consider removing everything that appears after the word 'study'.

The manuscript is well written but there are some grammar errors that can be fixed by a proof-reader. There are also many phrases/words that are redundant. For example, this sentence 'Regarding distribution, achieving effective drug penetration through the blood-brain barrier (BBB) is challenging, particularly for treating central nervous system (CNS) disorders due to tightly-packed endothelial cells, which are practically impregnable.' Authors did not number the pages hence I can't refer you to the page! I am not sure what information the authors want to convey. Are you referring to distribution of the drug or achieving drug penetration?

There are a many other similar grammar errors in the manuscript that need to be fixed to avoid ambiguity.

Reference to the figures and tables in the text: the author has used lower case in cases when referring to figures and tables. Please check and be consistent. For example: Table 1 should appear as Table 1 and NOT table 1!

The figures that depict the docking poses are not very clear. It is not possible to see the text in the figures. Either increase the size and show the images A, B, C one below the other. Or improve the clarify of the image.

The figure captions can be corrected as well. For example it figure captions should read as follows: Figure 3 : Two-dimensional (2D) representation of the docking poses in the active site of AChE (PDB ID:1EVE) for compounds A) C23, B) C24 and C) the reference

Figures 9-13 that has the structures showing the docking, are also not very clear. Again, improve the clarity of these images.
